# A CuNi-Loaded Porous Magnetic Soft Material: Preparation, Characterization and Magnetic Field-Controlled Modulus

**DOI:** 10.3390/ma15041412

**Published:** 2022-02-14

**Authors:** Jingyuan Bai, Xuejiao Wang, Meilin Zhang, Jin Zhang, Xiaolin Chen, Yanan An, Renguo Guan

**Affiliations:** 1 School of Materials Science and Engineering, Northeastern University, Shenyang 110819, China; baekkyungwon@163.com (J.B.); xuejiaowang0213@163.com (X.W.); zhangmeilin9696@163.com (M.Z.); 2 Engineering Research Center of Continuous Extrusion, Ministry of Education, Dalian Jiaotong University, Dalian 116028, China; cxiaolin@djtu.edu.cn; 3 State Key Laboratory of Solidification Processing, Center of Advanced Lubrication and Seal Materials, Northwestern Polytechnical University, Xi’an 710072, China; anyanan@nwpu.edu.cn

**Keywords:** magnetic soft composite, CuNi, porous matrix

## Abstract

Novel porous magnetic soft materials (pMSMs) based on a poly (vinyl alcohol) (PVA) porous matrix filled with CuNi nanoparticles (NPs) of around 70 nm were synthesized. Initially, magnetic CuNi NPs were fabricated by the reduction of Ni and Cu ions with hydrazine hydrate in ethylene glycol medium in the absence of other capping agents. The pMSMs are subsequently fabricated by mixing CuNi NPs and PVA through freezing-drying process. The as-obtained pMSMs can respond to a magnetic field, i.e., the compressive modulus increase under a magnetic field of 0.23 T. The experimental results indicate that CuNi NPs can easily move to form chain-like structures under the application of a magnetic field. A combination of direct observation and finite element modeling has shown that under the influence of a magnetic field, chain-like aggregates of CuNi NPs lead to self-reinforcement of the pMSMs and, thus, to the increased compressive modulus. From a technological point of view, these materials with good magnetic responsiveness and moderate mechanical strength have potential applications in artificial muscle, soft actuators and drug release, to name a few.

## 1. Introduction

Magnetically responsive soft composites (MSCs), composed of magnetic components fixed in highly elastic polymer matrices, demonstrate promise in a wide variety of fields, such as soft robotics [1,2], sensors [3], biomedical devices [4], vibration control [5], etc. They integrate the unique magnetic-responsive properties coming from the filler and good mechanical performance provided by the polymer. In particular, this combination of properties within the MSMs leads to the appearance of some unique phenomena, such as change of mechanical behavior under an action of an external magnetic field and the shape memory effect [6,7,8,9]. However, magnetic phases are fixed in the rather tough polymer, which will to some extent decrease the magneto-mechanical coupling effect.

During the past few years, porous magnetic soft materials have been prepared to further study the magneto-induced modulus change with lower matrix toughness, and to enable them to suit the properties of the system for intended application. For example, a flexible porous system based on ethylene propylene diene rubber (EPDM) containing magnetic carbonyl particles were successfully prepared using a commercial foaming agent [10]. This porous system exhibits lower mechanical hysteresis compared to its non-porous counterpart. Yu [11] and coworkers demonstrated that porous MSCs (pMSCs) greatly impact stress–strain relationships and are more sensitive to external magnetic field. Moreover, for their range of applications to be extended further, the preparation of high porosity and well-interconnected pore network can provide additional and unexpected functionalities. Well-connected pores allow the drugs infiltration and cell attachment, which can be utilized for biomedical purpose [12,13]. The porous structure can also be used for the construction of a slippery surface by filling them with inert lubricants [14]. The pores can even open and close through an externally controllable on-off operation of given magnetic field. Chen [15] et al. studied the controllable delivery of therapeutic drugs by precisely controlling the mechanical movement of intelligent porous ferrogel under a magnetic field. In this system, the magnetic properties were tuned by modifying the particle size of the embedded Fe_3_O_4_. Thus, large particle sizes are favored, since strong saturation magnetization (*M_s_*) and low coercivity (*H_c_*) offers good “magnetic-sensitive effect”. However, large particles may block the pores and eventually decrease the drug uptake. MSMs based on magnetic metallic nanoparticles (NPs) can provide the optimal *M_s_* and *H_c_* by simply tuning the NPs composition.

Binary CuNi system has attracted the attention of researchers for many years due to their electrocatalytic and antifouling properties, high tensile strength, as well as good corrosion resistance [16,17,18]. Ni-rich CuNi alloys (i.e., Ni content greater than 61 at%) are known to be ferromagnetic [18]. Within the ferromagnetic range, the CuNi magnetic properties vary with the composition, particles’ size and distribution. It is worth mentioning that, although the CuNi-polymer composite materials have already been studied to some extent, those of CuNi-based MSMs remain unexplored. Compared to magnetite, Cu-Ni offers the advantage that the *M_s_* and *H_c_* can be tuned by adjusting the Ni content in the alloy. For the aforementioned reasons, it is envisaged that the development of CuNi MSMs combining large porosity and tailored magnetic response emerges as a challenging engineering subject that could be appealing for applications such as magnetically actuated micro/nanoelectromechanical systems (MEMS/NEMS), oil-water separation, self-lubricated system or even robotic system.

In this work, we report on a freeze-casting method, under mild condition, to fabricate porous MSMs based on poly (vinyl alcohol) (PVA) filled with well-defined CuNi NPs. A highly porous scaffold loaded with CuNi NPs can be obtained by this freezing-dry method, i.e., CuNi-PVA composites. These composites with different CuNi loading were prepared and the specific surface area, morphology, magnetic and mechanical properties were evaluated in detail. It was shown that mechanical behavior can be controlled through an application of an external magnetic field.

## 2. Materials and Methods

### 2.1. Reagents and Materials

Commercial NiCl_2_·6H_2_O (Aladdin Chemistry Co., Ltd., Shanghai, China), CuCl_2_·2H_2_O (analytical purity, Sinopharm Chemical Reagent Co., Int, Beijing, China), ethylene glycol (analytical purity, Sinopharm Chemical Reagent Co., Itd, Beijing, China), N_2_H_4_·H_2_O (Tianjin bodi Chemical Reagent Co., Ltd., Tianjin, China) and PVA (Aladdin Chemistry Co., Ltd., Shanghai, China) were used as received.

### 2.2. Synthesis of CuNi NPs

NiCl_2_·6H_2_O (1 M, 107.2 μL), CuCl_2_·2H_2_O (1 M, 42.8μL) and ethylene glycol (30 mL) were mixed first and stirred at 500 rmp in a 50 mL round-bottom flask. Then, the mixture was heated up to 100 °C, followed by the addition of 1 mL N_2_H_4_·H_2_O. After the solution turned colorless, the reactor was cooled naturally in laboratory air. The products were collected and washed three times with ethanol and water, respectively, and then vacuum dried at 50 °C for 8 h.

### 2.3. Fabrication of Porous CuNi-PVA Composites

In order to maintain a good dispersion in PVA, a varying amount of CuNi NPs (40 mg, 60 mg, 80 mg) NPs were first washed with 20 mg/L PVA aqueous solution for three times. The PVA-treated NPs were then collected by a strong magnet and mixed with 1 mL 8% PVA solution in a sample vial (Φ 16 mm × 26 mm). The vial was left in a freezer at −40 °C for 4 h. Finally, the frozen sample was freeze-dried at the same temperature for 48 h.

### 2.4. Characterization

Scanning electron microscopy (SEM) images and energy-dispersiveX-ray spectroscopic (EDX) analyses were performed on a TESCAN microscope operated at 5 kV. X-ray diffraction patterns of the MMFs were recorded with a PANalytical B.V. X-ray diffractometer in the 39–56° and 10–80° 2*θ*-range (step size = 0.1°, step time = 2.4 s) using Cu K_α_ radiation (λ = 0.154178 nm). Room temperature hysteresis loops were collected using a vibrating sample magnetometer (VSM) from Oxford Instruments (PPMS-9), with a maximum applied magnetic field of 2 T. Nitrogen adsorption-desorption isotherms were measured at 77.3 K by using a Micromeritics Trister 3000 system (ASAP 2460). The samples were degassed at 60 ℃ for 5 h on a vacuum line. The standard multi-points Brunauer–Emmett–Teller (BET) method was used to calculate the specific surface area. The pore size distributions of the materials were derived from the adsorption branches of the isotherms based on the Barett–Joyner–Halenda (BJH) model. Mechanical tests were performed using a universal test machine (Instron model 3282). Magnetic field was applied during the compressive test using two NdFeB magnets of 230 mT. The magnetic field was measured with a Tesla Meter (YHT TD 8620).

## 3. Results

In order to fabricate porous CuNi-PVA composites, CuNi NPs were first synthesized by a solution-based “one pot” method. The Cu and Ni precursors were reduced by N_2_H_4_ in the presence of ethylene glycol (EG) at 100 °C. EG functions as the reaction medium in this system and as a protective layer on the particle surfaces, preventing them from agglomeration. Initially, when introducing nickel and copper chloride into EG, the solvation of the metallic species by EG molecules occurs. Upon the addition of hydrazine hydrate into the system, EG ligands are substituted by N_2_H_4_ ligands [19,20]. Several complexes can be formed in the solution, such as Ni(Cu)(N_2_H_4_)_2_Cl_2_, Ni(Cu)(N_2_H_4_)_3_Cl_2_ and Ni(Cu)(N_2_H_4_)_6_Cl_2_, which act as precursors for the final production of CuNi NPs. As the mixture is heated, the excess (uncomplex) hydrazine reduces the dissolved metallic species, leading to the formation of CuNi seeds.

**Figure 1:**
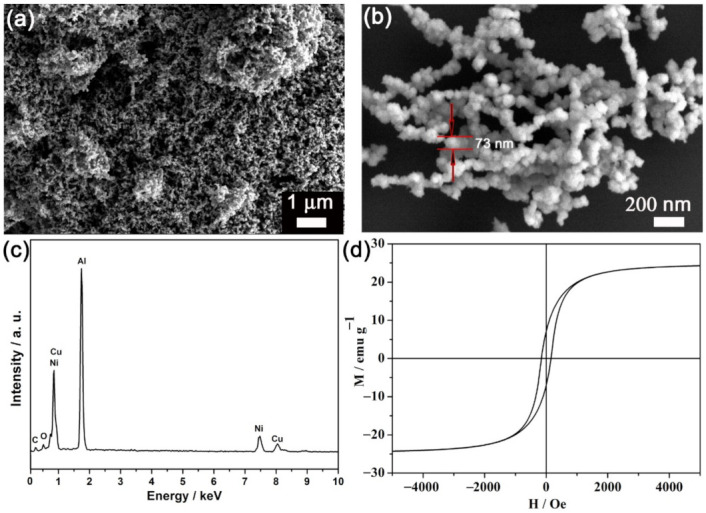
(**a**) SEM image, (**b**) zoomed SEM image, (**c**) EDX spectrum, (**d**) room temperature hysteresis loop of the as-prepared CuNi NPs.

Shown in Figure 1a is the panoramic view of the product morphology. The micrograph indicates that the large quantity and good uniformity were achieved using this “one pot” strategy. These NPs exhibited a roughly spherical shape with a mean particle size of around 70 nm (Figure 1b). The corresponding EDX spectrum (see Figure 1c) shows that the composition was around Cu_30_Ni_70_, which is in accordance with the molar ratio of the agents added. The aluminum signal in Figure 1c came from the aluminum foil onto which the CuNi NPs were drop-casted. A very low oxygen and carbon signal were detected as well, indicating that the as-obtained CuNi NPs survived the washing process. The magnetic hysteresis loop of the as-prepared CuNi NPs is exhibited in Figure 1d. The coercivity (*H*_c_) was found to be around 150 O_e_.

**Figure 2:**
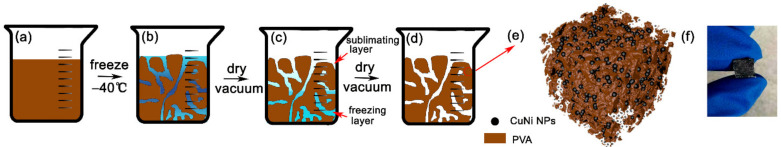
Schematic illustration of the fabrication process: (**a**) preparation of the composite mixture; (**b**) freezing the mixture at −40 °C; (**c**,**d**) freezing-dry process; (**e**) cross-linked network of CuNi-PVA; (**f**) cross-sectional photographic image of the cylindrical CuNi-PVA (80 mg CuNi NPs).

The procedure for the preparation of porous CuNi-PVA composites by the freeze-drying process is shown in Figure 2. Briefly, CuNi NPs were dispersed in PVA aqueous solution and immediately frozen at a temperature of −40 °C for 4 h. Pores were generated subsequently by removing the ice phase during freeze-drying. The drying process initials from the upper surface of the sample and proceeds toward the body. The voids left by the first sublimated ice will serve as extravasating channels for the following gas-water. Therefore, the scaffold obtained by this method exhibit an irregular porous structure with relatively good pore interconnectivity. Figure 2e depicts the porous network formed by PVA, incorporating with CuNi NPs in its structure. The sample was removed from the container after being freeze-dried and cut with a knife to observe the cross-section. As shown in Figure 2f, the CuNi-PVA sample was a rather homogeneous blackish color, indicating that the CuNi NPs was uniformly distributed within the PVA skeleton.

**Figure 3:**
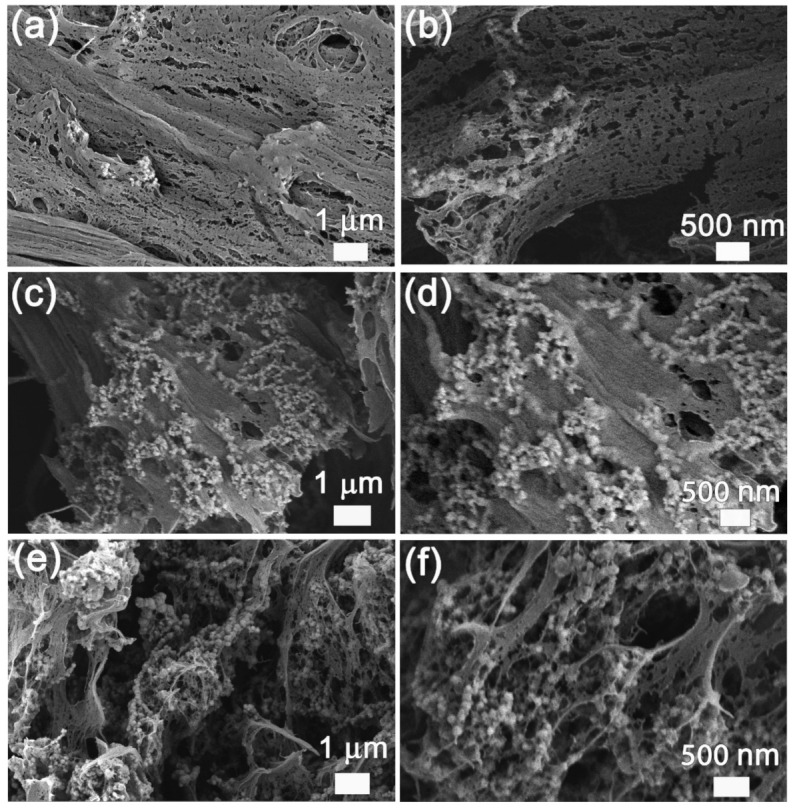
Cross-sectional SEM images at low and high magnifications of the CuNi-PVA composites with different CuNi loading amount: (**a**,**b**) 40 mg; (**c**,**d**) 60 mg; (**e**,**f**) 80 mg.

When preparing the pMSMs, a large loading amount of magnetic filler is required to generate a strong magnetic responsiveness, but it also leads to an increase in stiffness, which, in turn, will decrease the overall strain. When the PVA concentration was made at 8%, porous CuNi-PVA with different NP loadings were prepared, as shown in Figure 3. In our laboratory, attempts to produce porous CuNi-PVA composites with a PVA concentration greater or lesser than 8% failed. In fact, CuNi NPs aggregated and settled down quite fast in diluted PVA solution. While an obvious decrease in porosity can be observed in the sample prepared with a PVA concentration of 10% (see Figure S1). On the contrary, highly porous architecture with hierarchical porosity was shown throughout the scaffold of the sample prepared with 8% PVA solution and large voids were often observed.

These pores were shaped by the ice crystals during the freeze-drying process. Therefore, the loading amount exerted little influence the porosity of the resultant CuNi-PVA composites. Clearly, all these composites possess hierarchical porosity, with pore sizes ranging from nanometer to micrometer. CuNi NPs are linked to the network with little sedimentation taking place. When the CuNi uptake was 40 mg (donated as CNP-40), NP clusters could be found with large average distances between the neighboring magnetic regions. Due to the small loading amount, only a few NPs can be observed on the cross section, shown in Figure 3a. With the increasing loading amount to 60 mg (CNP-60) and 80 mg (CNP-80), the distribution of NPs became rather homogeneous and the neighboring magnetic clusters distance became smaller. The pore size distributions and specific surface area of the three samples were characterized by nitrogen gas sorption and are exhibited in Figure S2. The three samples show type IV isotherms with a sharp capillary condensation step at high relativel pressure (P/Po = 0.8–0.9) as well as H1 type of hysteresis loops [21,22]. These observations suggest that the obtained porous composites possessed relatively large pore sizes. As summarized in Table 1, the addition of NPs into PVA slightly enlarges the average pore width from 159.2 nm to 193.0 nm. The surface area values fluctuate with the change in the loading amount. These slight changes can be ascribed to the experimental uncertainty, owning to the fact that calculation results are largely dependent on the absorption/desorption time and temperature. Combining the BET results and SEM images, the specific surface area of the CuNi-PVA composites prepared by this method was around 25 m^2^ g^−1^, independently of the CuNi NPs uptake. 

**Figure 4:**
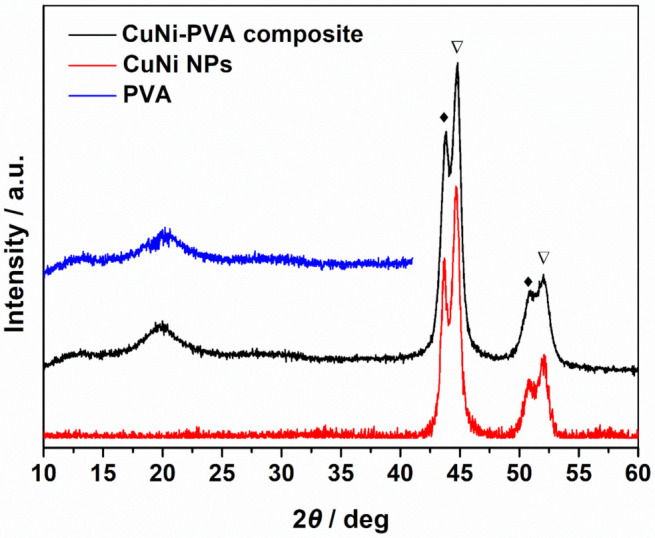
XRD patterns of CuNi NPs, CuNi-PVA composites and PVA. Peaks donated by ◆ and ▽ belongs to Cu-rich and Ni-rich phases, respectively.

The crystallographic structure and phase composition of CuNi NPs and the CNP-60 were studied by XRD (Figure 4). As for CuNi NPs, six diffraction peaks corresponding to Cu (111), Ni (111), Cu (200), Ni (200), Cu (220) and Ni (220) reflections of face-centered cubic (fcc) structure were detected. The significant peak splitting indicates that phase separation occurred during the reduction process. The position of the Ni (111) reflection was shifted ca. 0.07° toward lower angles compared to the tabulated position (44.481). Likewise, the position of Cu (111) reflection shifted the same value toward higher angles with respected to the tabulated position (43.342°). Hence, the NPs consisted of Cu-rich and Ni-rich phases, i.e., some Ni was dissolved into α_1_ Cu phase, while some Cu was dissolved into α_2_ Ni phase). In fact, the phenomenon of phase separation occurred quite commonly during the preparation procedure for the CuNi system. The different reduction potential and nucleation rate accounts for the phase separation. Besides, according to the local environment model, the total free energy of the system is lower when Ni atoms segregate to form Ni-rich magnetic cluster [23,24]. By comparing the XRD pattern of CNP-60 and the CuNi NPs, all the peaks corresponding to Cu- and Ni-rich phases can be straightforwardly indexed. Hence, the freezing-drying process did not introduce any extra phases nor change the crystallographic structure of CuNi. In addition, PVA is a well-known crystalline polymer [25,26], and the crystalline nature originates from the strong intermolecular interaction between PVA chains through hydrogen bonds. In our case, one broad diffraction peak centered at 19.4°, corresponding to the spacing of (101), represented the semi-crystalline feature of PVA. This means that the as-obtained PVA contained a mass of free voids between the polymeric chains in the amorphous phase, where the CuNi NPs can be trapped [27]. However, the low diffraction intensity suggests that cross-linking PVA with CuNi leads to the decrease in the intermolecular interaction between PVA chains and, thus, to the crystalline degree. To explore the interaction between the CuNi NPs and PVA in the composite, XRD of the pure porous PVA fabricated through the same process was performed. Compared with the CuNi-PVA composite, the XRD pattern of PVA demonstrated no occurrence of new peaks but did demonstrate a slightly blue shift. This means that the grafted CuNi NPs did not change the basic structural configuration of PVA and were well-encapsulated inside the polymeric metrics.

**Figure 5:**
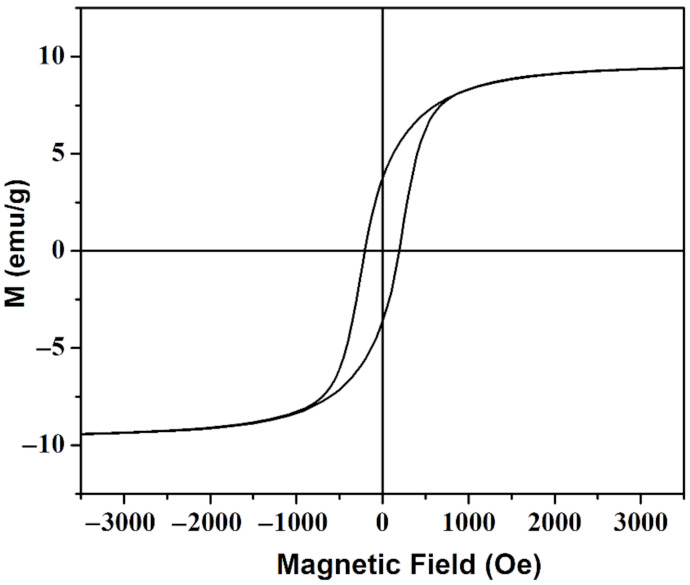
Room temperature hysteresis loop of the as-prepared CNP-80.

The magnetic property of sample CNP-80 was measured and shown in Figure 5. The sample presents a well-defined hysteresis loop with *M_s_* of 9.35 emu/g and *H_c_* of around 207 Oe. The slight increase in *H_c_* compared to that of the CuNi NPs was due to dipolar interactions that weakened cooperative reversal of the ferromagnetic clusters and tended to increase *H_c_*. When applying an external magnetic field, the relatively low *H_c_* render the CuNi nanoparticles easily demagnetized and remagnetized to the direction of the applied magnetic field. Moreover, the obtained values are comparable with that of the as-reported PVA-Fe_3_O_4_ magnetic hydrogel fabricated through freezing-thawing method [15], in which a magnetic sensitive behavior is observed. When applying a magnetic field to CNP-80, a strong magnetic field can be induced in the porous composite, thus, modifying its mechanical property.

## 4. Discussion

**Figure 6:**
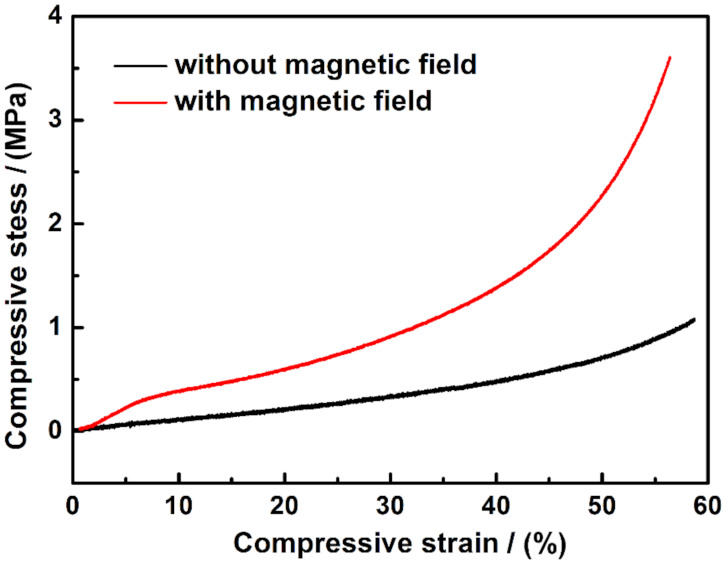
Compressive Stress-strain curves of CNP-80 with and without magnetic field.

The compressive testing of CNP-80 with and without applied external magnetic field was performed using a universal test machine. A magnetic field was applied during the compression process using two horizontal NdFeB permanent magnets. The sample was placed in between the two paralleled magnets, as shown in Figure S3. In such a way, a uniform magnetic field was applied perpendicularly to the sample during the test. The initial position of the upper compression plate is exhibited in grey; along with the compression direction, the upper fixture moved downwards to compress the sample. As PVA-based CNP-80 is much softer compared to the rigid magnets, the modulus change induced by NdFeB is neglected in this work. The compressive strain–stress curve in the absence and presence of the magnetic field is shown in Figure 6. As seen from the slope, when no magnetic field is applied, compressive stress gradually increased with the strain, suggesting elastic deformation. Notice that, with a strain up to 60 %, CNP-80 is still elastic. The compressive behavior under external magnetic field involves the magnetic dipole-dipole force and internal stress of the PVA matrix. The embedded NPs retain a certain mobility, owing to the porous nature of the scaffold and the physical adsorption of CuNi to PVA. Under an applied homogeneous magnetic field, the magnetic CuNi particles with the porous scaffold tend to align into chains. As a result, the rearrangement of the magnetic NPs increases the possibility of internal deformation of the porous PVA. The magnetic fillers changed their position with respect to the initial state when no magnets were placed, until a new equilibrium state was reached. Due to dipolar interaction, the magnetic CuNi NPs chain up, suggesting a decrease in the average particle distance along the formed chains. Therefore, the increase in elastic modulus can be ascribed to the increase in mechanical stiffness along the chain which acts as a reinforcement.

**Figure 7:**
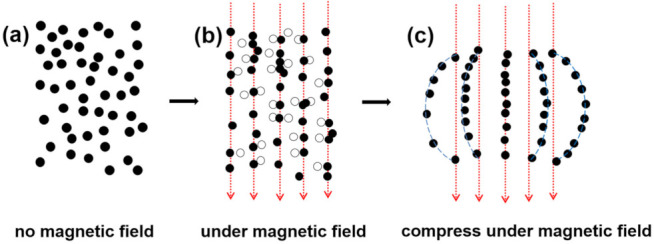
Schematic illustration of magnetic CuNi NPs displacements: (**a**) initial position; (**b**) under the action of external magnetic field, (**c**) under the action of external magnetic field and compression.

Shown in Figure 7a is the initial position of the magnetic NPs, in which random distribution can be expected. When sandwiching the sample with two magnets, the intensity of the magnetic field is fixed, so as to induce the magnetic moment of the NPs and their dipolar interaction forces. In the absence of external deformation, i.e., no compressive force is applied, the NPs fall into an equilibrium state to build up the columnar structure within the polymer matrix. As demonstrated in Figure 7b, the open circles represent the initial position of NPs, and they align with the external magnetic field. At small deformation, this architecture is only slightly disturbed, so that every particle stands as it was. A linear strain-stress output shows up in a small strain range of less than 6% in Figure 6, suggesting the elastic behavior. When increasing the compressive force, the chains were curved, and the amplitude of bend increased from the middle of the sample to the edge. As a result, the distance between neighboring NPs changed, along with the compressive modulus. With an increase in strain, the non-linear behavior becomes more pronounced (Figure 6).

**Figure 8:**
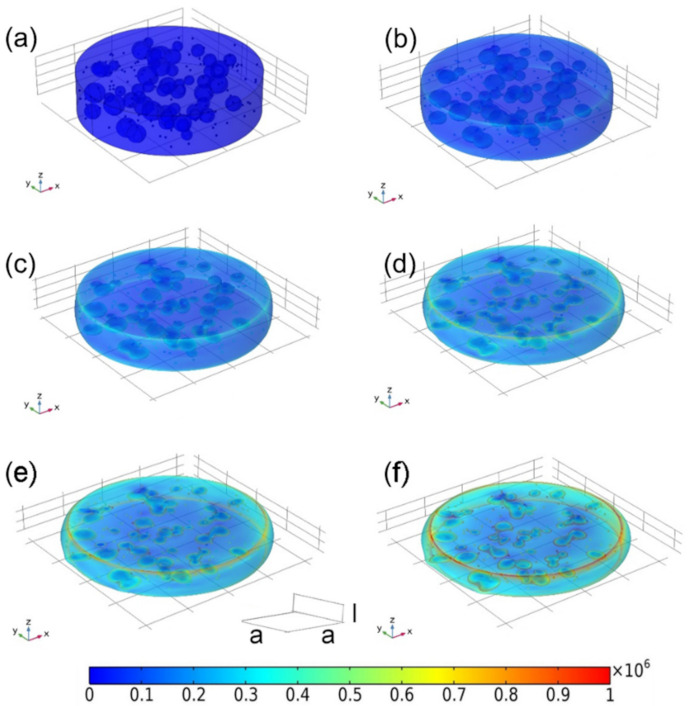
Schematic portrayal of finite element model in a compression process without the action of external magnetic field. The deformation degree is (**a**) 0%, (**b**) 20%, (**c**) 30%, (**d**) 40%, (**e**) 50%, (**f**) 60%. The rainbow bar indicates the compressive stress (unit MPa).

**Figure 9:**
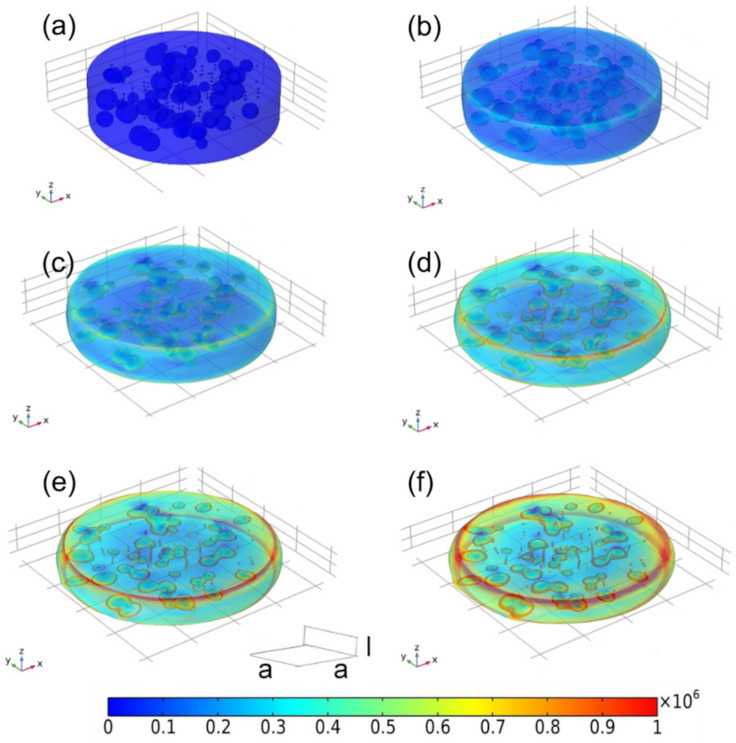
Schematic portrayal of finite element model in a compression process with the action of external magnetic field. The deformation degree is (**a**) 0%, (**b**) 20%, (**c**) 30%, (**d**) 40%, (**e**) 50%, (**f**) 60%. The rainbow bar indicates the compressive stress (unit MPa).

The compressive stress distribution of the as-prepared CuNi-PVA composite with and without external magnetic field were analyzed by chain-like model [28]. For the sake of similarity, we assume that the CuNi NPs have a spherical shape of diameter *R*, pores are globular shape with varying sizes and the porous matrix has a cylindrical architecture of diameter *4a* and height 4*l*. To some extent, this theoretical porous model can be approximated as a small portion taken from the obtained CuNi-PVA composite. The stress under a given amount of deformation with and without external magnetic field is estimated by finite element method. Figure 8a shows the original model of the as-prepared CuNi-PVA composite, in which the NPs are randomly distributed. Observation from Figure 8b-f suggests that the compressive stress gradually increase with strain. And the pore inside the matrix are flattened and elongated. Under a magnetic field, the CuNi NPs tend to align in the direction of the field, and the densification process is illustrated in Figure 9. At the first glance, the behavior of the deformation is quite similar, in which the compression of the pores and the increase in the cylindrical diameter can be detected. However, it appears that, under the same deformation condition, the specimen resulted in higher stress change upon applying a magnetic field. The simulated stress–strain curves obtained for random oriented and aligned samples are shown in Figure S4. One can see that, under the influence of the magnetic field, the compressive stress increased; in the range of large deformation (40–55%), the stress rises faster. This result is in good agreement with the experimental curves, as shown in Figure 6.

**Figure 10:**
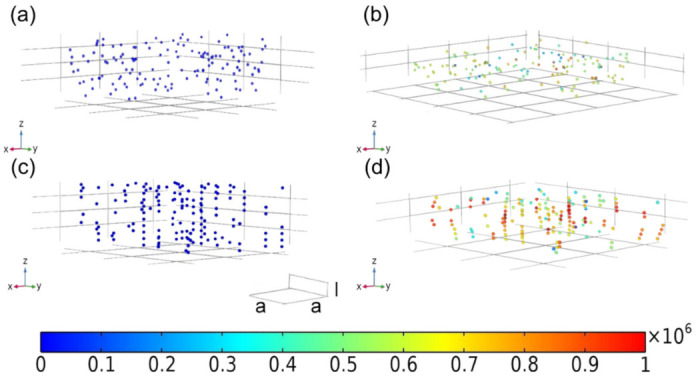
Stress distribution on NPs with (**c**,**d**) and without (**a**,**b**) the action of external magnetic field. (**a**,**c**) before compression, (**b**,**d**) after compression. The rainbow bar indicates the compressive stress (unit MPa).

The porous matrix was further removed in order to observe the strain distribution on the CuNi NPs and influence of the chain bending on the magnetic-induced modulus [29]. As shown in Figure 10a,b, the randomly oriented arrangement of the NPs remained after the compression. The spacing between the particles along the compression direction was reduced, while the value was increased in the vertical compression direction. NPs with chained arrangement tended to bend after compression (Figure 10c,d). The closer to the boundary of the material matrix, the greater the radian of the bended chain [30]. In addition, the stress of these NPs was obviously greater than that of the randomly oriented ones. A similar phenomenon, reported elsewhere [31,32], was observed by researchers. The chained NPs demonstrated pronounced magnetic response when they became curved, which will, in turn, prevent the further bend over. As the NPs are trapped inside the polymeric matrix, the whole system appears with higher modulus. Moreover, the average distance between NPs along the formed chain reduces, resulting in an increase in mechanical stiffness, owning to the high rigidity of the CuNi NPs with respect to the PVA matrix. As shown in Figure 10d, instead of forming continuous long chain, short chains with same orientation are obtained due to the limited loading amount of the NPs. The magnetic response of these short discontinuous chains is surely lower than that of the long chain, yet still higher than randomly distributed NPs. In view of their moderate mechanical strength, good processability and quick responsiveness, the as-obtained materials are expected to be suitable candidates for magnetic field offer potential applications in drug delivery, artificial muscle and soft actuators.

## 5. Conclusions

In summary, physically cross-linked porous PVA composites containing CuNi NPs as magnetic filler were prepared through a freeze-dry process. It has been proven that the specific surface area of the CuNi-PVA composites prepared by this method is independent of the CuNi uptake. Under the action of a magnetic field, some rearrangement of the CuNi NPs within the composites takes place. It is conjectured that chain-like aggregates of magnetic NPs formed, resulting in self-reinforcement of the soft materials and, thus, considerable increase in its modulus. Compared to commonly used magnetically responsive fillers. The magnetic properties of CuNi can be tuned with the composition, particles’ size and distribution. For the envisioned application, CuNi with a high value of *M*_s_ and low value of *H*_c_ were favored, as this would secure magnetic maneuvering and, thus, the enhanced modulus. This property, together with the porosity, makes this material a promising candidate for widespread technology applications, such as self-sensing soft actuators, micro-sensors, magnetic separators and drug delivery.

## Figures and Tables

**Table 1:** Physical properties of the as-prepared porous CuNi-PVA composites.

Sample	Surface Area (m^2^/g)	Average Pore Width (nm)
CNP-40	29.2	159.2
CNP-60	23.2	187.8
CNP-80	25.1	193.0

## Data Availability

The data presented in this study are available on request from the corresponding author.

## Supplementary Materials

The following supporting information can be downloaded at: https://www.mdpi.com/article/10.3390/ma15041412/s1, Figure S1: Low (a) and high (b) magnification SEM images of CuNi-PVA composites with PVA concentration of 10%, Figure S2: Nitrogen adsorption/desorption isotherm (left figure) and pore size distributions (right figure) of porous CuNi-PVA composites with different NPs uptake: (a) 40 mg, (b) 60 mg, (c) 80 mg. Adsorption isotherms are shown by blue lines with solid squares and desorption isotherms are illustrated by red lines with open squares, Figure S3: Illustration of experimental setup for investigating the compression modulus property of CNP-80 with NdFeB permanent magnets, Figure S4: Simulated compressive strain-stress curves of the CuNi-PVA composite.
