# Supplementary material for: A CuNi-Loaded Porous Magnetic Soft Material: Preparation, Characterization and Magnetic Field-Controlled Modulus
Source: Materials (Basel). 2022 Feb 14;15(4):1412. doi: 10.3390/ma15041412 (PMC8877354; doi:10.3390/ma15041412)
Supplement: Supplementary file 1 [file materials-15-01412-s001.zip › materials-1577638-supplementary.pdf]

materials

Article

A CuNi-Loaded Porous Magnetic Soft Material: Preparation, Characterization and Magnetic Field-Controlled Modulus

Jingyuan Bai [1], Xuejiao Wang [1], Meilin Zhang [1], Jin Zhang [2,3,*], Xiaolin Chen [2,3], Yanan An [3] and Renguo Guan [1,2,*]

[1] School of Materials Science and Engineering, Northeastern University, Shenyang 110819, China; baekkyungwon@163.com (J.B.); xuejiaowang0213@163.com (X.W.); zhangmeilin9696@163.com (M.Z.)
[2] Engineering Research Center of Continuous Extrusion, Ministry of Education, Dalian Jiaotong University, Dalian 116028, China; cxiaolin@djtu.edu.cn
[3] State Key laboratory of Solidification Processing, Center of Advanced Lubrication and Seal Materials, Northwestern Polytechnical University, Xi'an 710072, China; anyanan@nwpu.edu.cn
* Correspondence: jinzhang@djtu.edu.cn (J.Z.); guanrenguo@sina.cn (R.G.)

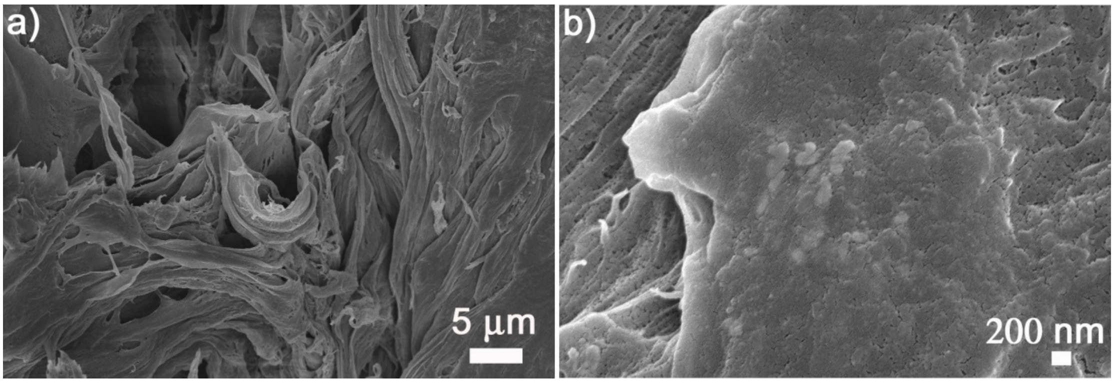

Figure S1. Low (**a**) and high (**b**) magnification SEM images of CuNi-PVA composites with PVA concentration of 10%.

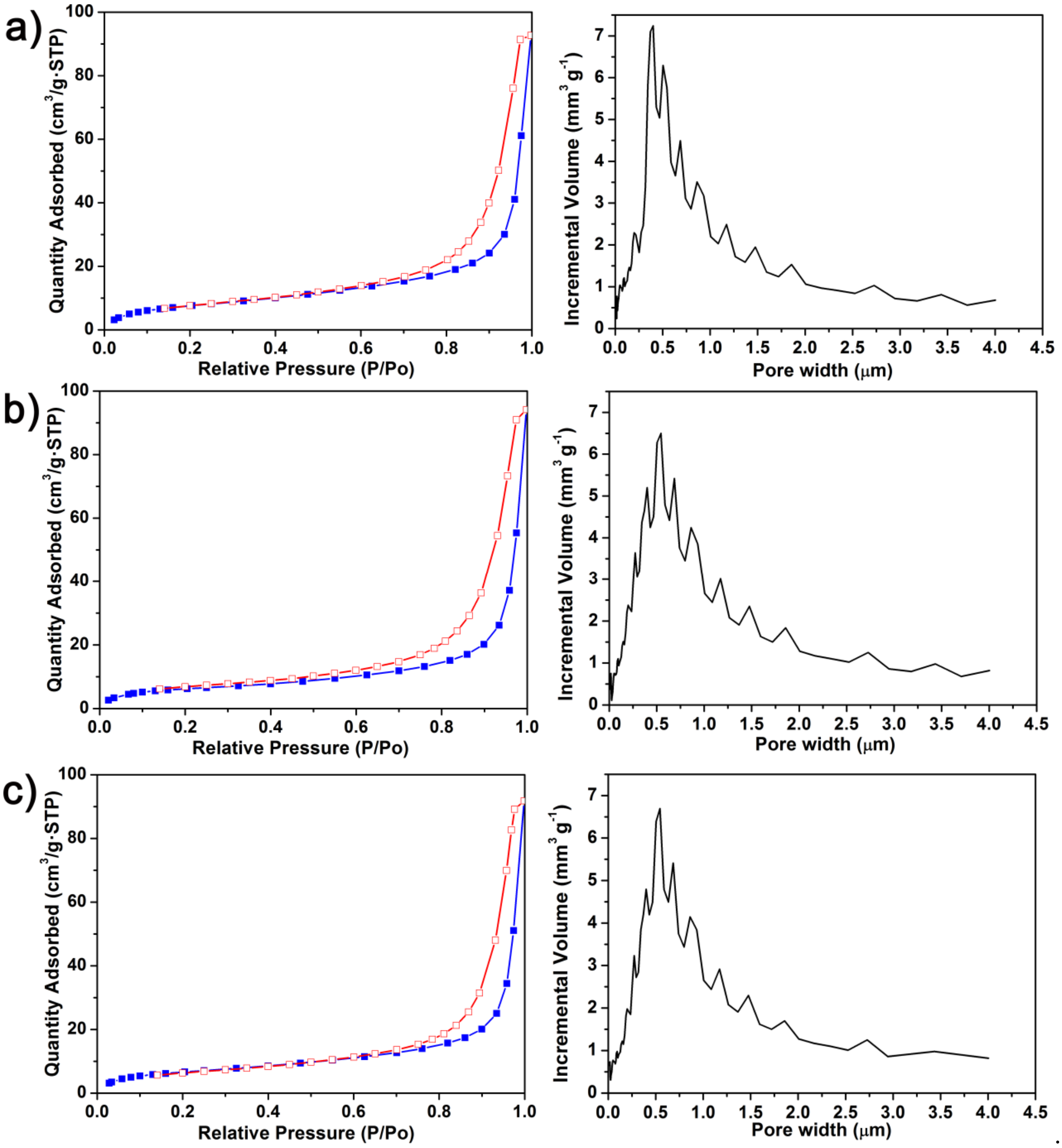

Figure S2. Nitrogen adsorption/desorption isotherm (left figure) and pore size distributions (right figure) of porous CuNi-PVA composites with different NPs uptake: (**a**) 40 mg, (**b**) 60 mg, (**c**) 80 mg. Adsorption isotherms are shown by blue lines with solid squares and desorption isotherms are illustrated by red lines with open squares.

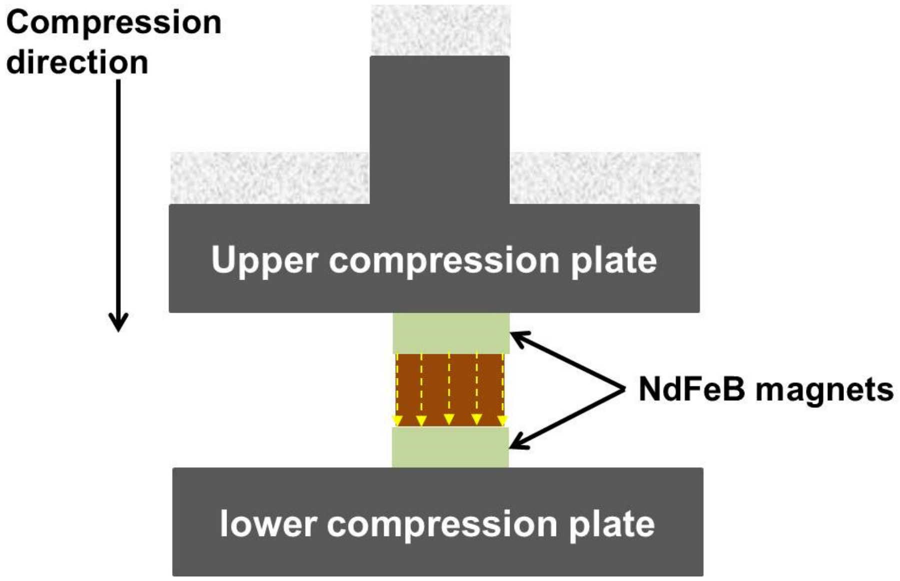

Figure S3. Illustration of experimental setup for investigating the compression modulus property of CNP-80 with NdFeB permanent magnets.

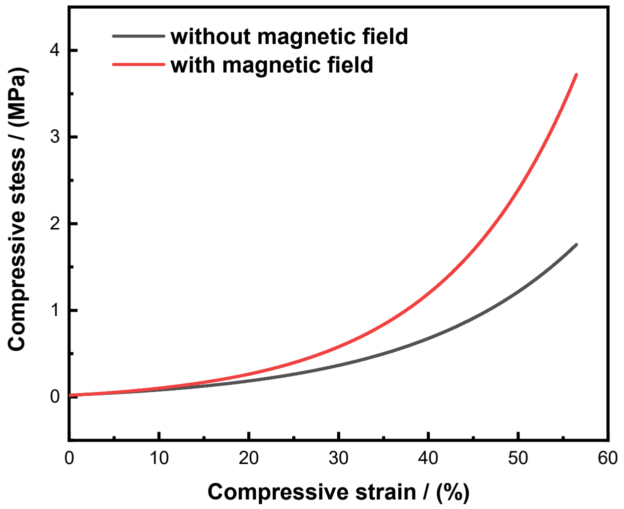

Figure S4. Simulated compressive strain-stress curves of the CuNi-PVA composite.
